# The Construction of British Columbia's Palliative Care Benefits Policy and Implications for Nursing Practice

**DOI:** 10.1177/15271544241298656

**Published:** 2024-12-09

**Authors:** Carly Elliott, Helen Brown, Lydia Wytenbroek, Farinaz (Naz) Havaei

**Affiliations:** 1 School of Nursing, University of British Columbia–Delta, Delta, British Columbia, Canada; 2 UBC School of Nursing, University of British Columbia, Vancouver, British Columbia, Canada

**Keywords:** palliative care, health policy, critical discourse analysis, delivery of health care, Canada, clinical decision-making

## Abstract

Nurses have a critical role to play in creating, implementing, critiquing, and advancing health policy within diverse contexts to ensure people living with life-limiting illness receive equitable and ethical access to palliative care services and programs. This article describes a critical analysis of the British Columbia's Palliative Care Benefits (BCPCB). The BCPCB is a provincial government program in British Columbia (B.C.), Canada, that provides eligible residents with palliative care services at home. Utilizing Fairclough's Dialectical–Relational Critical Discourse Analysis, the study investigates the process by which BCPCB determines B.C. residents’ eligibility and access to resources proportionate to their need, function, illness burden, and urgency. This article reviews the construction of palliative care in Canada and how current biomedical perspectives construct palliative care policy and services in B.C. The findings indicate that the BCPCB program produces vague, discriminatory, and ableist prognostication practices through the implementation of its eligibility policy. This article also suggests that palliative care nurses are optimally positioned for policy influence: to critique, disrupt, and transform the BCPCB Program and palliative care practices.

## Introduction

As healthcare practitioners, nurses can assist patients with the navigation of complex and often inaccessible services, policies, and legislation. Since nurses maintain the ethical obligation to promote and ensure equitable care for all—and are the largest health professional workforce in Canada that spends the greatest amount of time in closest proximity with patients—nurses are ideally positioned to advocate for the incorporation and implementation of equity-oriented care into nursing programs, practice settings and broader health systems (McGibbon et al., 2014). Therefore, it is crucial for nurses to be aware of and to lobby against discourses of inequity underlying and being enacted through policy.

Dominant biomedical discourses not only construct our understandings of life-limiting illness and palliative care, but also the frameworks for addressing them (Fairclough et al., 2012). Discourses function to establish literary bodies in hierarchical relations to each other in accordance with dominant power relations; thus, discourses within policies maintain power to shape, influence, and establish equitable treatment (Fairclough et al., 2012). However, discourses also play a role in legitimizing and formalizing inequitable treatment (Fairclough et al., 2012). It is therefore important for nurses to critically analyze the discourses that give rise to particular policies and the healthcare context to maintain or improve social systems of inequity for people experiencing life-limiting illness (Fairclough et al., 2012). Life-limiting illness refers to an active, progressive, or advanced disease, that has little or no prospect of cure and that is likely to result in the death of the person in the coming days, months, or years (Fassbender et al., 2005).

This study explored how British Columbia's Palliative Care Benefits (BCPCB) Registration Policy and the *Home and Community Care Policy Manual* (Policy Manual) texts shape nursing practice and produce power relations that hinder equitable access to palliative care benefits for British Columbian residents living with life-limiting illness in their homes. This study aimed to contribute nursing knowledge for palliative care practice and policy to: (1) illuminate broader political and economic factors that influence BCPCB and Home and Community Care policy development and implementation (2) and contribute to developing nursing practice and policy actions to improve population health and reduce health inequities for clients living with life-limiting illnesses.

## Background

According to the Canadian Institute for Health Information (CIHI, 2022), British Columbia (B.C.) averages over 45,000 deaths per year with the three most common underlying causes of death being cancer (25%), diseases of the circulatory system (17%) and diseases of the respiratory system (10%). CIHI (2022) estimates that only 52% of these decedents in B.C. were formally registered as palliative care patients despite most of these deaths being related to chronic, life-limiting illnesses rather than a sudden death event. One study found that people in B.C. prefer to die at home or in the community yet 53% of British Columbians still die in the hospital every year (Jayaraman & Joseph, 2013). While some of these hospitalizations may be necessary or unavoidable, especially in the final days of life, there is a need for greater access to quality palliative and end-of-life care earlier in an individual's illness trajectory (CIHI, 2022).

### History of Palliative Care in Canada

The conditions of life and death remained largely unchanged throughout much of twentieth-century Canada (Risse & Balboni, 2013). Death remained an integral part of daily life: epidemics, war, accidents, infection, and childbirth claimed most Canadians’ lives (Curtis & Mcgee, 2000). Apart from war- and accident-related mortalities, most people in the early 1900s died at home and were cared for by family members and friends (Aries, 1975). By the 1930s, medical advances (such as immunization and antibiotics) and public health efforts resulted in the reduction of deaths from infectious diseases and a shift to chronic illnesses as the number one cause of death ((Hetzler & Dugdale, 2018).

The period of sustained economic prosperity that followed World War II led to dramatic changes in life and death in Canada. As government resources grew, so too did public funding for healthcare (Vatovec et al., 2013). By 1950, the location of both birth and death had shifted from home to hospital in Canada ((Hetzler & Dugdale, 2018). Both the public and the medical profession were coming to believe in the powers of science and medicine to control and cure illness (McCue, 1995). Bolstered by advances in medical science, physicians and nurses focused increasingly on curative measures, which led to perceiving death as a “medical failure” (Amundsen, 1978). This shift in attitudes toward death in Canada was the beginning of the medicalization of dying (Aries, 1975).

Medicalized dying refers to when the dying process is facilitated or prolonged by medical intervention (Amundsen, 1978). Management of near-death patients came to be focused almost exclusively on arresting or reversing their near-fatal conditions in harmony with the basic disease orientation of biomedicine (Amundsen, 1978). In Canada, as in many parts of the world, society was fixated on cure rather than a peaceful death, which led to increased funding for hospital research and helped nurture a national psychology in which death was seen as an unacceptable outcome that should be prevented at all costs (Fulop, 1985).

Hospice and palliative care services first emerged in Canadian hospitals in the 1970s in response to the challenges brought about by the medicalization of dying, the staggering economic concerns from the overutilization of medicines and technology, and an increase in cancer diagnoses among the Canadian population (Canadian Hospice and Palliative Care Association, 2023). By the 1990s, the Canadian government took several measures to address the ever-expanding health care budgets and ballooning deficits threatening all levels of government (Risse & Balboni, 2013). The Canadian government's neoliberal approach to manage economic deficits resulted in areas across Canada experiencing deinstitutionalization of health care services, including the closure of small community hospitals, and the merger and specialization of urban hospitals (Risse & Balboni, 2013). In all areas of health care, deinstitutionalization effectively meant that patients were sent home or onto the streets (Risse & Balboni, 2013). In the absence of adequate home care services and no longer being able to rely upon the support of an extended family or community network, immediate families (especially women in families) and informal caregivers began to experience the crushing demands of caregiving without any compensation (Risse & Balboni, 2013).

#### BCPCB Policy

The BCPCB program was implemented in 2001 and was created in response to the changing demographic of B.C. residents and the projected increase in economic deficits in health care (Crooks, 2009). An increasingly aging population living with chronic illness coupled with the challenging topography of B.C. required a strategy to improve access to palliative care services in the home setting and thereby reduce medicalized dying in hospitals (Crooks, 2009). BCPCB is a provincially funded social service program in B.C. that offers coverage of symptom management medications through the PharmaCare BC Palliative Care Drug Plan (Plan P), and coverage of medical supplies and equipment through local B.C. health authorities for eligible clients (British Columbia Ministry of Health Services, 2021). BCPCB is available to B.C. residents of any age who have reached the end stage of a life-threatening disease or illness (i.e., a prognosis of 6 months or less) and wish to receive palliative care services at home (British Columbia Ministry of Health Services, 2021). A client's eligibility and enrollment in BCPCB is determined by a physician's assessment that deems a client to be living in the last 6 months of life (British Columbia Ministry of Health Services, 2021). In addition, clients must agree to receive palliative care services in their home and must consent to comfort care as the sole focus of treatment to qualify for BCPCB (British Columbia Ministry of Health Services, 2021). Once a physician deems a client eligible for BCPCB, a palliative care nurse assesses the individual in their home to determine the level of care and resources they are eligible for under the BCPCB program (British Columbia Ministry of Health Services, 2019). The level of care and resources that a BCPCB client is eligible for is determined by the palliative care nurse's assessment, which is based on a client's need, function, urgency, and illness burden as outlined in the British Columbia Ministry of Health Services' (2013a, 2019) *Home and Community Care Policy Manual: Home health services*.

The implementation of the Pharmacare “Plan P” benefits program under BCPCB improved B.C. residents’ abilities to receive palliative care services in the home by providing pharmaceuticals, but it also marked a shift in government support from free-standing hospices to in home-based palliative care options. This policy reform confirms the influence of neoliberal political values that supported this shift to de-institutionalize hospice and palliative care to the home.

After the BCPCB program's implementation, key stakeholders (i.e., formal and informal caregivers for individuals living with life-limiting illness) identified obstacles and challenges to equitable access to BCPCB for B.C. residents (Crooks, 2009). Eligibility criteria defined by BCPCB registration require B.C. residents to have a prognosis of <6 months and to agree to forgo curative treatment options to be considered eligible (Fishman et al., 2009). These eligibility criteria place individuals with life-limiting illnesses in a difficult position of having to make a choice between continuing active treatments and accessing BCPCB services (Fishman et al., 2009). Requiring a decision to pursue or decline treatment to access BCPCB can result in barriers to timely palliative care service utilization, especially in marginalized populations (Fishman et al., 2009). Research has suggested that cultural and religious values, as well as historical and systemic traumas, can significantly influence the decision of individuals to continue or forgo treatment (Fishman et al., 2009). For example, Indigenous clients may find it difficult to decline futile or aggressive treatment if they have a sense of mistrust due to a shared collective history of colonialism (Kelly & Minty, 2007).

Prognostication by physicians is another significant factor that is operating to determine eligibility for BCPCB (British Columbia Ministry of Health Services, 2021). General practitioners are the primary physicians who complete BCPCB registration for B.C. residents (British Columbia Ministry of Health Services, 2021). Prognostication requires a comprehensive assessment of the client, which can be challenging for general practitioners: many clients with life-limiting illnesses are too weak to attend clinic appointments and general practitioners generally do not have the capacity to make home visits for client assessments due to time and fiscal constraints (McCallan & Daudt, 2021). Some general practitioners will defer BCPCB registration to another physician who has been more actively involved with the client, which is most often an oncologist. This tendency to defer to another physician has contributed to the trend of individuals with cancer having greater access to BCPCB than individuals with non-malignant diagnoses (Fitzsimons et al., 2007). The CIHI (2023) found that patients with cancer were the most likely to be identified as palliative (77%) while patients with dementia were the least likely to receive palliative care in the last year of life (39%). This inequity in palliative care access among individuals with life-limiting illness of differing diagnoses suggests that the current BCPCB policy criteria do not accurately capture nor adequately support the end-of-life experience of all B.C. residents (Rix et al., 2014).

The biomedical values of affective-neutrality, efficiency, detachment, and rationality pre-eminently underlie the operation of the current Canadian health care system (Moller, 2018). With the biomedical emphasis on diagnosis and treatment of physical dysfunctions, palliative care providers are focused on the “surveillance and control of the process of dying” (Floriani & Schramm, 2012). Framing life-limiting illness as an empirical field of professional knowledge and expertise leads to medical professionals being put in the role of gatekeeper (Rioux & Daly, 2019). In this role, access to BCPCB and its associated resources is assessed by determining a person's needs and provision of resources based on criteria evaluating their range of “independent functional ability” and “illness burden” as specified in the language of the BCPCB policy and Policy Manual (Rioux & Daly, 2019). However, this approach is entirely empirical and does not account for structural inequities that could impact health, well-being, and prognosis, such as income, social status, housing, education, physical environments, coping skills, and social support networks (Florani & Schramm, 2012). Assessment through observation also does not capture the client experience—this requires entering the client's psyche, history, culture, beliefs, and social relations, and interpreting the meaning of what the person has lived or is living through (Mino & Lert, 2005). When medical practitioners are positioned as gatekeepers to BCPCB and its associated resources, biomedical and functional approaches to illness are privileged in palliative care policies, programs, and services, and little power is left for clients and families to determine when and how to access palliative care services at home (Rioux & Daly, 2019).

### Palliative Care Nursing Practice

The first author undertook this master's thesis study during a period when revisions were underway within the Framework of Palliative Care in Canada Act (Health Canada, 2017). The Framework's goal was to improve access for Canadians to palliative care services at all levels of care, including hospitals, home care, long-term care facilities, and residential hospices (Health Canada, 2017). In 2022, when this study was undertaken, no review or evaluation had been conducted in relation to palliative care service in Canada; specifically, how those related health policies and procedural documents impact BCPCB access. This study was undertaken to investigate how the BCPCB and the Policy Manual texts shape nursing practice and produce power relations that hinder equitable access to palliative care benefits for people living with life-limiting illness in their homes.

## Methods

### Theoretical Approach

Conceptualizations of life-limiting illness and hospice palliative care are enmeshed within the culture, histories, and societies in which they occur, and are therefore, inextricably bound to the political concerns, norms, and values of the specificities of place, culture, and society (Roberts, 2005). Critical social theory (CST) provided the theoretical foundation to guide the critique of power relations constructed through critical analysis of existing discursive texts within the policies that determine eligibility for BCPCB and the allocation of associated resources to eligible clients.

Critical discourse analysis (CDA) is defined as a theory that analyzes and exposes structural relationships of dominance, discrimination, power, and control as manifested in language (Fairclough, 1992). In other words, CST assists CDA to explore and expose how the structural context of oppression, dominance, and inequality are enacted and reproduced through textual features within a social and political context (Evans-Agnew et al., 2016). CDA guided the analysis while CST supported the structural analysis of oppression, dominance, and inequality embedded in eligibility criteria and associated resource allocation for BCPCB (Fairclough, 2003).

Foucault et al.’s (1977) claim about the inseparability of power and knowledge in discourse also theoretically guided this study, where both act as a form of social control through societal institutions and ideology. Foucault et al. (1977) suggest that institutions organize knowledge-producing communities and thereby shape the production of discourse and knowledge, all of which is framed by ideology. Discourse and ideology work in tandem to influence social action through communicative practices (Purvis & Hunt, 1993). This theoretical framework and empirical analysis method from Foucault (1977) was utilized to bring to light historical and modern forms of power and how these emerged in language to influence palliative care policies.

Fairclough's (2009) Dialectical–Relational CDA focuses analysis on relations among varying elements (such as power, values, and beliefs) as dialectical. The four iterative stages for data collection and analysis included: (1) selection of the *social wrong* as research topic; (2) naming obstacles to address the social wrong (e.g., identified through the analysis of dialectical relations between semiosis, texts, and other social elements); (3) interdiscursive and linguistic/semiotic analysis of texts; and (4) identification of how to move beyond obstacles.

The social wrong that is the focus of this thesis is the manner in which BCPCB and the Policy Manual removes self-determination from B.C. residents by granting unequivocal power to social actors who already occupy dominant social positions, such as physicians, and nurses. Thus, the analysis shows how BCPCB, and the Policy Manual exert power in the direction of dominant and discriminatory relations of power that deepens social inequity.

Obstacles to address the social wrong were identified in dominant discourses within the BCPCB program and the *Home and Community Care Policy Manual* and further explored. Obstacles include the social and historical influences toward the conceptualization of palliative care, biomedicalism, ableism, and the positioning of people with life-limiting illness alongside the history of colonization and increasing neoliberal orientations of the Canadian government (Ashcroft et al., 2013). In this stage of analysis, the biomedical justifications for eligibility to BCPCB were critiqued and deconstructed, as well as the functional ability assessment practices within the Policy Manual that act discursively to determine how resources are allocated to clients within the BCPCB program.

It was imperative to view and analyze the BCPCB through a postcolonial lens. Postcolonialism brings critical academic study to the cultural, political, and economic legacy of colonialism and imperialism, focusing on the impact of human control and exploitation of colonized people and their lands (Seto Neilsen et al., 2013). The privileging of biomedical discourse in BCPCB and the Policy Manual is continuous with the cultural, political, and economic legacy of colonialism and imperialism; specifically, how deinstitutionalizing palliative care services to the home occurred without consideration of the consequences of colonialism. For example, Indigenous people within Canada face challenges navigating and accessing palliative home care services due to unique historical, social, cultural, economic, and systemic factors (Castleden et al., 2010). For rural, small-town, and on-reserve Indigenous populations, these challenges can be compounded by geographic remoteness, limited infrastructure and resources, lack of Indigenous home care workers, relocation-related challenges, and lack of culturally appropriate services (Hotson et al., 2004). The long-standing colonial history that Indigenous peoples in Canada contend with is a strong cause for distrust of the mainstream health care system and Western medicine (Rix et al., 2014). This distrust makes it difficult to form clinician/family partnerships in care planning and ultimately affects the illness trajectory, symptom management, and support available for Indigenous peoples at end of life (McGrath, 2006).

A textual analysis of selected sections from the BCPCB Registration Policy (2021) and selected chapters of the British Columbia Ministry of Health Services’ (2013a, 2019) *Home and Community Care Policy Manual* was undertaken. These texts were analyzed using Fairclough's (2009) Dialectical–Relational CDA and were selected based on their authority and influence over accessibility to BCPCB and its associated resources (British Columbia Ministry of Health Services, 2015). The British Columbia Ministry of Health Services' (2015) BCPCB Registration policy was chosen for analysis because it exerts power; it is the primary document used to interpret and determine eligibility for BCPCB for B.C. residents. The British Columbia Ministry of Health Services’ (2013a, 2019) *Home and Community Care Policy Manual: Home health services* data was also included in the analysis because it is the other primary document used to interpret, implement, and allocate BCPCB resources into practice for registered nurses, and into the lives of B.C. residents and their families. Relevant and seminal scholarly articles, as well as gray literature from the British Columbia Ministry of Health Services' library, that define BCPCB eligibility, implementation, resource allocation, and evaluation of these policies, were also included.

## Results

The analysis engaged the socio-historic events and forms of discrimination impacting the current conceptualization of life-limiting illness and palliative care services in B.C. The findings reflect a CDA of how these events and forms of discrimination produce and reify the discursive context under which the BCPCB and the Policy Manual were created and how these texts shape nursing practice and produce power relations that hinder equitable access to palliative care benefits for B.C. residents living with life-limiting illness in their home.

### Obstacles to Address the Social Wrong

Populations that experience the greatest disparities in access to BCPCB are primarily groups that have experienced systemic marginalization and discrimination (CIHI, 2023). B.C. residents living in rural communities have been found to have higher rates of chronic illness and disability as well as lower socioeconomic status compared to urban populations (Rix et al., 2014). The CIHI (2023) found that palliative clients living in rural areas were more likely to be hospitalized primarily for palliative care compared with those living in urban areas (36% and 29%, respectively) and were also more likely to die in hospital than those in urban areas (29% and 23%, respectively). Geographic distance and remoteness are factors that influence access to palliative home care services and the quality of palliative home care available to clients (Rix et al., 2014). Eligibility criteria for BCPCB pose a barrier for some clients since eligibility requires a physician assessment, and physicians are often unavailable in rural areas (Rix et al., 2014).

With 14% of B.C.'s Indigenous populations living in rural areas, many Indigenous peoples must leave their communities if they wish to access palliative care services (CIHI, 2023). However, most Indigenous individuals wish to receive palliative care services at home and do not want to relocate (Rix et al., 2014). Not only are current palliative home care services geographically distant from rural communities, but they are often culturally unsafe for Indigenous peoples due to differences in language, values, beliefs, and expectations (CIHI, 2023). For elders who have survived the residential school system, a threat of re-institutionalization may also be a determining factor in accessing palliative care (Castleden et al., 2010). Other concerns in accessing palliative care services relate to needing to navigate the unfamiliar, loss of an established local support system and control, cultural alienation, limited access to cultural comforts and foods, financial burdens, communication challenges, and discomfort with Western medicine (British Columbia Ministry of Health Services, 2021, p. 2; McGrath, 2006). These contextual and institutional barriers ultimately hinder access to the resources and services offered by the BCPCB program for Indigenous peoples in B.C. living with life-limiting illness in their homes.

#### Dominant Values Exerting Impact

Postcolonial theory brings to light the cultural categorizations that develop through the privileging of certain social groups and their knowledge (Seto Neilsen et al., 2013). These categories are juxtaposed as favorable and unfavorable stereotypes that may act to reinforce the perceived superiority of the Canadian biomedical model (Seto Neilsen et al., 2013). Cultural categories used in health care are not neutral in that categorizations tend to normalize Western biomedical beliefs and problematize health beliefs and practices outside of this model (Seto Neilsen et al., 2013). For example, Indigenous cultural practices are often not reflected nor valued in Canada's biomedical and empirical health care culture (McGrath, 2006). Different perspectives on pain and side effects in palliative care can create challenges for Indigenous clients and families to navigate end-of-life care. For example, BCPCB offers “100% coverage of prescribed and over the counter palliative medications” (British Columbia Ministry of Health Services, 2021, p. 1) when a client is registered for the BCPCB program. However, certain palliative care pain management practices (i.e., the use of opioids for pain that causes drowsiness as a side effect) can interfere with the cultural tradition of the passing of oral knowledge of a dying loved one, which can result in culturally unsafe care practices (McGrath, 2006).

The resources that are offered to eligible clients under the BCPCB policy also reflect biomedical and postcolonial values by only including subsidized services that provide pharmaceutical or medical intervention, or assistive equipment to promote physical and functional independence (British Columbia Ministry of Health Services, 2021). The resources included in the BCPCB program do not include any supports that address emotional, social, financial, psychological, cultural, or spiritual needs (British Columbia Ministry of Health Services, 2021). Alternative or complementary modalities of treatment are also excluded from the resources offered by BCPCB (British Columbia Ministry of Health Services, 2021). Without consideration or support of health practices outside of the biomedical and empirical model, BCPCB benefits some populations of people over others based on cultural or personal values, resulting in unequal allocation and distribution of subsidized services at end-of-life to all B.C. residents. By privileging biomedical models of care, the discourse in BCPCB has embedded discrimination in the services and resources that it offers to B.C. residents with life-limiting illness.

### Analysis of Texts

#### Prognostic Complexities and BCPCB Eligibility

In 2021, the BCPCB policy was updated for physicians to utilize the Supportive and Palliative Care Indicators Tool (SPICT) instead of the Palliative Performance Scale (PPS) to determine life expectancy for individuals with life-limiting illness (British Columbia Ministry of Health Services, 2021). The SPICT replaced the PPS to provide a more holistic assessment of general poor health for determining prognosis rather than the diagnosis-specific language used in the PPS for prognosis determination (British Columbia Ministry of Health Services, 2021). The SPICT includes multiple categories of indicators, including “general indicators of poor health” as well as “clinical indicators of life-limiting illness” (British Columbia Ministry of Health Services, 2021, p. 2). An individual must have “at least two general indicators and one clinical indicator” (British Columbia Ministry of Health Services, 2021, p. 2) to be considered eligible for BCPCB. The specific language used in the BCPCB policy and SPICT assessment of “general indicators” for “poor or deteriorating health” include: “poor performance status with limited reversibility … dependency on others for care due to increasing physical and/or mental health problems … the person's caregiver needs more help or support … persistent symptoms despite optimal treatment of underlying condition(s) … the person (or family) asks for palliative care … chooses to reduce, stop or not have treatment … or wishes to focus on quality of life” (British Columbia Ministry of Health Services, 2021, p. 2). The SPICT in the BCPCB policy further categorizes “clinical indicators” into various illness groups, including: “cancer … dementia/frailty … neurological disease … heart and vascular disease … respiratory disease … kidney disease … liver disease … and other conditions” (British Columbia Ministry of Health Services, 2021, p. 2). Each category lists further details for conducting a clinical assessment, including observations of “decline in physical and functional ability (e.g., mobility, ability to eat, ability to swallow, ability to complete ADL's, incontinence, cognition/memory) … an inability to receive treatment due to frailty, severity of illness or medical futility … an increase in infections or febrile episodes, falls, or physical symptoms (e.g., pain, breathlessness)” (British Columbia Ministry of Health Services, 2021, p. 2).

The SPICT is completed by the physician after their general client assessment; this assessment includes a discussion with the client, family, and informal caregivers; a physical assessment; as well as a review of the client's medical history and other clinical investigations (British Columbia Ministry of Health Services, 2021). Although the client's and their family's and caregivers’ experiences and choices are considered in the physician assessment (e.g., the client's desire to “receive palliative care services at home”), clients and their families/caregivers do not have any decision-making authority specified in the policy language for registration for BCPCB (British Columbia Ministry of Health Services, 2021, p. 2). The utilization of the SPICT for prognosis determination is thereby limited in its regard of client and family/caregiver choice in consideration for eligibility for registration for BCPCB and limits client self-determination in care.

Also, the BCPCB's priority in determining program eligibility, as detailed in the policy, focuses on a “decline in physical or functional ability … an inability to receive treatment due to … severity of illness … [and] increase in … falls, physical symptoms” (British Columbia Ministry of Health Services, 2021, p. 2). The BCPCB does not include criteria for social, emotional, spiritual, financial, and psychological needs and deficits, and does not consider the impacts of colonization and systemic oppression. This focus on observable factors for assessment as detailed in the BCPCB policy discourse—and its absence of other less visible factors—for determination of BCPCB brings to light the privileging of biomedical, empirical and rational discourse in the determination of eligibility for BCPCB and how this discourse omits consideration of the needs of systemically underrepresented and vulnerable residents of B.C. that are living with life-limiting illness, including populations experiencing homelessness, poverty, social isolation, mental illness, systemic racism and historical trauma.

#### Discourse Shaping Nursing Practice

Once deemed eligible and registered for the BCPCB program by a physician, individuals with life-limiting illness are assessed in their home by a palliative home care nurse to determine eligibility for publicly subsidized services (British Columbia Ministry of Health Services, 2019). The *Home and Community Care Policy Manual* is the document that outlines criteria for client eligibility, and palliative home care nurses use this Policy Manual to determine the level of resources an individual registered for BCPCB can receive (British Columbia Ministry of Health Services, 2013b, 2019).

Health conditions are defined in the Policy Manual, in the context of eligibility for subsidized home and community care services, as when the individual has “chronic health conditions that impair the individual's ability to function independently … has health conditions requiring care at home rather than hospitalization … or requires end-of-life care for a life-limiting condition” (British Columbia Ministry of Health Services, 2013b, Ch.2.B, p. 4). The focus of these criteria is on functional ability, independence, and the ability to manage the health condition at home suggesting embedded biomedical and neoliberal values (British Columbia Ministry of Health Services, 2013b). By only focusing on functional ability, the physical aspects of the assessment process dominate consideration of eligibility, with little consideration of emotional, social, psychological, cultural, or spiritual aspects of care needs. In addition, the determination of eligibility based on the ability to manage a health condition in the home serves the neoliberal agenda of the Canadian government to shift provision of care from institutions to the home to contain publicly subsidized health care costs and ultimately to privatize end of life care (Bjornsdottir, 2009).

Although palliative home care nurses are given authority to allocate BCPCB resources by the discourse used in the Policy Manual, nurses are operating under the power of neoliberal managerial technologies created by the British Columbia Ministry of Health Services that are aimed at reducing health care expenditure (Bjornsdottir, 2009). This new technology rendered work previously performed by nurses, such as holistic palliative care practices that are centered on emotional, psychological, and spiritual support, invisible and therefore obsolete (Bjornsdottir, 2009). These technologies include overt rules, as defined in the Policy Manual discourse, that require the nurses to stress client and caregiver independence and “self-care” (British Columbia Ministry of Health Services, 2019, Ch.4.D., p. 1). Oudshoorn et al. (2007) observed the pressures palliative home care nurses receive from management to “cut costs” by finding the least expensive home-care services, reducing the frequency of their services, or recommending private pay services. Driven by neoliberal values of scarcity and efficiency promoted by the Canadian government, this directive emphasizes standardization while also seriously limiting the options available to palliative home care nurses to provide quality palliative care to clients in the home, as well as take variability in needs and wishes among home-care clients into consideration (Bjornsdottir, 2009). Without the ability to customize client care to the unique needs of individuals and their families, it becomes increasingly difficult for nurses to provide culturally safe and trauma-informed care that incorporates diverse values, beliefs, and expectations for palliative and end-of-life care (Rix et al., 2014). This results in inequitable palliative care access and services for historically marginalized groups, including people experiencing homelessness, poverty, immigrants, people of color, and Indigenous peoples (Rix et al., 2014).

The nursing knowledge and discourse used in the Policy Manual to conduct assessments for palliative care services continue to be grounded in biomedicalism and its philosophical premises in positivism (McGibbon et al., 2014). Despite integration of the language of “holism,” nursing science is based on positivist beliefs that the whole can be reduced to the sum of its parts (James & Field, 1996). This can be observed in the Assessment section of the Policy Manual in that nurses are reducing a client's experience with dying to identifying their “health conditions and functional status” (British Columbia Ministry of Health Services, 2013b, Ch.2.D, p. 1) while creating a care plan that maximizes the use of private pay “community services” and unpaid informal “caregivers” (British Columbia Ministry of Health Services, 2013b, Ch.2.D, pp. 1–2). This biological reductionism in assessment processes runs in tandem with empirical nursing theories and nursing diagnoses that further sustains conditions of social domination, limits client autonomy and responsibility, and oppresses individuals and groups, including clients and families who experience end-of-life symptoms that are not directly observable, such as social, mental, emotional, spiritual, and existential suffering. Privileging observable physical symptoms over invisible social, mental, emotional, systemic, and existential symptoms when assessing eligibility for BCPCB is a form of ableism (Mannor & Needham, 2024). Discrimination between groups to gain access to subsidized palliative care services results from viewing groups of individuals with diagnoses that have more invisible symptoms as “less deserving” of service than individuals with more observable and physically disabling symptoms because of their diagnoses (Mannor & Needham, 2024). Experiences of life-limiting illness and dying are inherently unique and individual, and profoundly influenced by physical, social, historical, economic, and psychosocial factors (Crooks, 2009). Assessment practices that only rely on observable symptoms and physical deficits will not provide equitable palliative care access to all B.C. residents that is inclusive of all life-limiting illness diagnoses.

### How to Move Beyond the Obstacles

The current social order can evolve to ensure the safeguarding of personal rights through promotion of self-determination and dignity, thereby helping to mitigate the use of healthcare provider gatekeeping. Nurses maintain the ethical obligation to promote and maintain self-determination for people living with life-limiting illness, including the need to create and advocate for more equitable palliative care policies in B.C.

## Discussion

Critical policy analyses for nursing practice that employs CDA can challenge and assist the transformation of current practices, policies, and legislation that may be reinforcing or contributing to inequity (Fairclough et al., 2012). The findings highlight how BCPCB eligibility is determined by physicians and nurses through the implementation of various assessment practices within the BCPCB and Policy Manual. Although the stated intentions of BCPCB and Policy Manual are to ensure the fair allocation of resources to people with life-limiting illness with the greatest need, these policies in fact contribute to *unfairness* in accessing and distributing BCPCB resources. The findings show how the BCPCB and Policy Manual disproportionately affects people experiencing numerous and intersecting forms of structural discrimination in its application. Therefore, if the BCPCB and Policy Manual are not serving all clients, the critical question remains as to whose interests and needs are being privileged and served.

Eligibility for palliative care services under the BCPCB program is constructed to primarily serve the government health authorities enforcing the legislation, versus the clients accessing BCPCB. Healthcare providers are obliged by health authorities to utilize problematic forms within the BCPCB and Policy Manual to govern their clinical decision-making for determining eligibility and equitable resource allocation of subsidized palliative care services (British Columbia Ministry of Health Services, 2013b, 2019, 2021). Collaboration with patients and their families within BCPCB and the Policy Manual for determining eligibility to BCPCB resources are minimal and further, are not obligated, when identifying clinical indicators of disease, diagnosis, prognosis, or appropriate service hours (British Columbia Ministry of Health Services, 2013b, 2018, 2021). All decision-making authority remains with the physician and nurse who are working in suboptimal working conditions within constrained health care systems.

### Palliative Care Nursing Practice

Palliative care nurses are positioned to work with competing models of care since broad conceptualizations of palliative care maintain biomedical orientations that reinforce individualism and paternalism. Conversely, the Canadian Nurses’ Association's code of ethics (CNA, 2017) guides nurses to provide care that promotes fairness, social justice, beneficence, and accounts for social contexts. Furthermore, the Framework for Palliative Care in Canada guides palliative nursing care practice nationally by outlining ethical principles of a palliative approach, including care that is holistic, client and family-centered, and focused on improving quality of life (Health Canada, 2017).

Implementation of inequitable assessment measures formalized under the Policy Manual positions nurses in contradictory ways—between professional obligations under the Policy Manual and ethical nursing practice (Canadian Nursing Association, 2017). To implement the Policy Manual, nurses are limited in their ability to incorporate a patient's right to make decisions, and in many circumstances, their voice (Bjornsdottir, 2009). This directly conflicts with the ethics promoted for and by nurses such as “providing safe, compassionate, competent and ethical care” while “promoting justice” (CNA, 2017, pp. 8–15). The BCPCB and Policy Manual further infringes upon various aspects of The Charter of Rights and Freedoms (Government of Canada, 1982), including sections of the right to liberty, the right not to be deprived thereof, and equality provision. Depriving people's rights to sufficient resources and caregiver supports for palliative care at home is morally distressing for nurses and traumatic for clients and their families (Bjornsdottir, 2009; Brazil et al., 2010).

### Recommendations for Policy, Nursing Practice, and Research

Legislation and palliative care policy must be reconstructed to comply with The Charter of Rights and Freedoms (Government of Canada, 1982) and the proclamation by the Standing Committee of the Canadian Senate (2000) that end-of-life care is the right of every Canadian citizen, balancing the “autonomy and liberty of the individual” with the need for palliative care services (Government of Canada, 1985). Both palliative care and human rights are based on principles of the dignity of the individual and the principles of universality and non-discrimination (Gwyther et al., 2009). BCPCB and the Policy Manual must be modified from their current forms to integrate opportunities for the voice of clients and their families to impact the assessment and decision-making process for eligibility determination of BCPCB and its associated resources; as well as expanding the definition of “end-of-life” beyond a prognosis of less than 6 months and utilizing validated assessment tools that incorporate psychosocial, economic and systemic factors to more accurately predict an individual's life span. Although expanding the definition of “end-of-life” care and further incorporating the voices of clients and families would likely increase the costs to the BCPCB and Home Care Nursing programs, research in Ontario has shown that home-based palliative care can save an estimated $4,400 per patient in health care costs and $191–$385 million annually if expanded to individuals currently not receiving such services (Canadian Society for Palliative Care Physicians, 2023). Proportional savings would be anticipated if similar programs were employed in B.C. (Canadian Society for Palliative Care Physicians, 2023).^1^

The reconstruction of the BCPCB and Policy Manual must also include a critical review and analysis of the various discourses relied upon that maintain problematic power relations of healthcare practitioners and individuals with life-limiting illness. Discourses of equity provide a counter to current health care practices and structures that rely on narratives and discourses based on physical deficit and functional ability. An equity orientation can also be applied to address varying forms of marginalization experienced by people with life-limiting illness, challenging the dominant socio-historic discourses in palliative care practice and legislation (such as biomedicalism, ableism, colonialism, and neoliberalism; Brennan, 2014). Although nurses operate within a neoliberal, biomedical, and positivist framework, nurses can engage in critical reflexivity, advocate for promote shared decision-making, and challenge discriminatory views while advocating for more equitable treatment for individuals with life-limiting illness in their workplace (Bjornsdottir, 2009).

Opportunities must be provided for the client, family, nurses, and interprofessional team to play a more significant role in the process of decision making for determination of eligibility for BCPCB and its associated resources. This could include expanding the role of the registered nurse (R.N.) to receive specialty education in illness progression, palliative care, prognosis determination, and using validated assessment tools to identify individuals who would benefit from palliative care services. If the BCPCB policy expanded the scope of the practitioner with registration authority to include specialty certified R.N.s, then R.N.s could register individuals with life-limiting illnesses for BCPCB as appropriate. This is particularly helpful in rural communities where access to palliative care resources and physicians is especially limited (Weng et al., 2022). Integration of an expanded R.N. scope into the interdisciplinary team of a community-based palliative care program would help to reduce professional practice barriers for nurses and has been shown to improve access to BCPCB registration, subsidized palliative care services and the quality of the dying experience for B.C. residents living in rural geographic locations (Weng et al., 2022).

Sustained research and data collection is also vital for long-term and continuous improvement in palliative care: population-level studies to investigate equity across client diversity factors for BCPCB registration would provide evidence needed to address current knowledge gaps (Health Canada, 2017). Studies that evaluate the predictive validity of the current SPICT used for assessment of BCPCB and its associated resources would provide insight into how to overcome current equity challenges and drive innovation toward effective tools and eligibility assessment practices (Health Canada, 2017). Another important future research study would include talking to palliative care patients and their informal caregivers about their experiences with the current BCPCB and Policy Manual and its implications to their health and well-being. To date, very limited research exists regarding ways nurses embody or resist inequitable or discriminatory palliative care policies and practices. Future research should seek to uncover how nurses, and healthcare practitioners more broadly, go about resisting or normalizing inequitable or discriminatory palliative care policies and practices.

## Notes

1. A more robust economic impact analysis was beyond the scope of the analysis given the research questions.
